# ALKBH5 promotes invasion and metastasis of gastric cancer by decreasing methylation of the lncRNA NEAT1

**DOI:** 10.1007/s13105-019-00690-8

**Published:** 2019-07-09

**Authors:** Jun Zhang, Shuai Guo, Hai-yan Piao, Yue Wang, Yue Wu, Xiang-yu Meng, Dong Yang, Zhi-chao Zheng, Yan Zhao

**Affiliations:** 1 Gastric Cancer Department, Liaoning Cancer Hospital & Institute (Cancer Hospital of China Medical University), No. 44 Xiaoheyan Road, Dadong District, Shenyang City, 110042 Liaoning Province People’s Republic of China; 2 Medical Oncology Department of Gastrointestinal Cancer, Liaoning Cancer Hospital & Institute (Cancer Hospital of China Medical University), No. 44 Xiaoheyan Road, Dadong District, Shenyang City, 110042 Liaoning Province People’s Republic of China; 3 Emergency Department, Sheng Jing Hospital of China Medical University, 36 Sanhao St, Heping District, Shenyang City, 110003 Liaoning Province China

**Keywords:** Long noncoding RNAs, Gastric cancer, Nuclear paraspeckle assembly transcript 1, Alkylation repair homolog protein 5, *N*^6^-Methyladenosine

## Abstract

*N*^6^-Methyladenosine (m^6^A) is the most common posttranscriptional modification of RNA and plays critical roles in cancer pathogenesis. However, the biological function of long noncoding RNA (lncRNA) methylation remains unclear. As a demethylase, ALKBH5 (alkylation repair homolog protein 5) is involved in mediating methylation reversal. The purpose of this study was to investigate lncRNA m^6^A modification and its role in gastric cancer (GC). Bioinformatics predicted interactions of ALKBH5 with lncRNAs. Five methods were employed to assess the function of nuclear paraspeckle assembly transcript 1 (NEAT1), including gene silencing, RT-PCR, separation of nuclear and cytoplasmic fractions, scrape motility assays, and transwell migration assays. Then, m^6^A RNA immunoprecipitation and immunofluorescence were used to detect methylated NEAT1 in GC cells. Rescue assays were performed to define the relationship between NEAT1 and ALKBH5. NEAT1 is a potential binding lncRNA of ALKBH5. NEAT1 was overexpressed in GC cells and tissue. Additional experiments confirmed that knockdown of NEAT1 significantly repressed invasion and metastasis of GC cells. ALKBH5 affected the m^6^A level of NEAT1. The binding of ALKBH5 and NEAT1 influences the expression of EZH2 (a subunit of the polycomb repressive complex) and thus affects GC invasion and metastasis. Our findings indicate a novel mechanism by which ALKBH5 promotes GC invasion and metastasis by demethylating the lncRNA NEAT1. They may be potential therapeutic targets for GC.

## Introduction

Gastric cancer (GC) is the most common malignant tumor of the digestive system. Although progress has been reported in terms of treatment, it is still the second leading cause of cancer-related death [16]. This high mortality is attributed to specific biological features of the disease, for instance, challenges related to timely diagnosis, delayed clinical manifestation, and high rates of invasion and metastasis [17]. Therefore, identification of biomarkers and their potential molecular mechanisms in GC is critical. With the completion of the Human Genome Project (HGP) and the parallel development of next-generation sequencing (NGS), human transcriptome analyses have revealed that over 98% of the transcriptional output encodes noncoding RNAs (ncRNAs). Evidence has confirmed that dysregulation of ncRNAs plays crucial roles in regulating oncogenes and tumor suppressor genes [15]. Long noncoding RNAs (lncRNAs), which constitute a class of ncRNAs mainly with lengths over 200 nucleotides with minimal evidence of protein-coding ability [7], have moved to the center of the ncRNA research arena. Researchers have demonstrated that lncRNAs affect and regulate gene expression at multiple levels, including the epigenetic, transcriptional, posttranscriptional, and translational levels. Although it has been reported that the lncRNA HOXC-AS3 [26], the lncRNA GClnc1 [17], and the lncRNA GMAN [30] are involved in the occurrence and development of GC, the potential mechanisms by which lncRNAs regulate GC need to be further explored.

*N*^6^-Methyladenosine (m^6^A) is the most common messenger RNA modification in eukaryotes. m^6^A-dependent mRNA regulation is crucial in mammals and affects diverse biological processes [4]. Its biological effect is mainly to regulate protein expression through “writers,” “erasers,” and “readers” [27]. This modification is reversible [6]. The classic “writer complex” catalyzes m^6^A methylation of mRNA and consists of core components including the m^6^A methyltransferases METTL3 (methyltransferase-like 3) and METTL14 (methyltransferase-like 14) as well as other regulatory subunits [10, 14]. Two well-known eraser enzymes, ALKBH5 (alkylation repair homolog protein 5) and FTO (fat mass and obesity-associated protein), are involved in mediating methylation reversal [6, 29]. Reader proteins recognize m^6^A-methylated transcripts and regulate pre-mRNA processing, translation, and degradation [21]. Given that RNA m^6^A modification is involved in gene expression regulation and various biological processes, we have reason to believe that abnormal m^6^A modification plays a vital role in the process of carcinogenesis. However, the mechanisms by which writers or erasers regulate lncRNA methylation require further investigation. Yang et al. [23] showed that m^6^A modification of linc-1281 regulates mouse embryonic stem cell differentiation. Decreases in KCNK15-AS1 methylation inhibit pancreatic cancer motility [5]. As m^6^A is a double-edged sword, understanding of its involvement in pathogenesis and tumorigenesis is still limited.

In this study, we demonstrated that the lncRNA NEAT1 (nuclear paraspeckle assembly transcript 1) and ALKBH5 were significantly overexpressed in GC and that high expression of NEAT1 and ALKBH5 was correlated with invasion and metastasis in GC. In addition, knockdown of ALKBH5 inhibited the expression of NEAT1 and upregulated the m^6^A level of NEAT1 RNA in GC.

## Materials and methods

### Cell lines and cell culture

The human normal gastric epithelial cell line GES-1 and the human gastric cancer cell lines SGC-7901 and BGC-823 were obtained from China Medical University (Shenyang, China). The cells were cultured in RPMI 1640 medium supplemented with 10% fetal bovine serum (FBS, Invitrogen, Carlsbad, CA, USA), 100 U/mL penicillin, and 100 μg/mL streptomycin (Invitrogen) at 37 °C under 5% CO_2_ and 1% O_2_. All experiments were repeated three times independently.

### Tissues and ethical statement

Fifty-seven GC tissues and matched nontumorous adjacent tissues were obtained from patients undergoing surgical resection at the Liaoning Province Cancer Hospital & Institute between 2015 and 2017. The patients signed informed consent before surgery at the Liaoning Province Cancer Hospital & Institute.

### Separation of nuclear and cytoplasmic fractions

According to the manufacturer’s protocol, total cellular fractions were divided into cytoplasmic fractions and nuclear fractions with a PARIS kit (AM1921; Thermo Fisher Scientific, Yokohama, Japan). The kit was able to isolate RNA from the same experimental sample and was guaranteed to separate the cytoplasmic and nuclear components before the RNA was separated.

### Real-time reverse transcription polymerase chain reaction

Total RNA from cells and tissue was isolated with TRIzol (Invitrogen) cell separation reagent according to the manufacturer’s instructions. A Promega cDNA core kit (Promega, Madison, WI, USA) was used to generate complementary DNA from 500 ng of total RNA. SYBR Master Mixture (Takara Bio, Inc., Kusatsu, Japan) was used for real-time polymerase chain reaction (real-time PCR) (LightCycler 480; Roche AG, Basel, Switzerland). Each sample was analyzed three times. U6 was used as a loading control. The fold changes in mRNA expression in different cells were determined by 2^-△△CT^ normalization. Each sample was analyzed in triplicate.

### Western blot analysis

SDS polyacrylamide gels (10–15%) and PVDF membranes were used to separate and transfer protein (20 μg) from cells, respectively. TBS-Tween buffer (20 mM Tris-HCl, 5% nonfat milk, 150 mM NaCl, and 0.05% Tween-20, pH 7.5) was used to block the membranes for 1 h at 21 °C after blotting. Then, the membranes were incubated with primary antibodies overnight at 4 °C (anti-ALKBH5, 1:200, Abcam; anti-EZH2, 1:200, Abcam; and anti-β-actin, 1:4000, Santa Cruz). Finally, the membranes were incubated with a secondary antibody (1:5000, Santa Cruz Biotechnology). β-Actin was used as a control. The gray values of the protein bands were measured with ImageJ (NIH, Bethesda, MD, USA). The averages of three independent measurements are presented as the final data.

### IHC

Both tumor and adjacent tissue sections (5 μm) were fixed on poly-L-lysine-coated slides. Antibodies were used to immunostain the sections (anti-*HIF-1α*, 1:100, Abcam, and *P4HB*, 1:50, Boster Biological Technology). Biotin-conjugated secondary antibodies were used for visualization. A VECTASTAIN ABC Elite kit (Linaris, Wertheim, Germany) and diaminobenzidine (DAB) substrate (Boster Biological Technology) were used for immunoperoxidase detection.

### Evaluation of IHC staining

Two experienced pathologists were responsible for evaluating the immunoreactivity of *HIF-1α* and *P4HB*. The staining intensity and the proportion of positive cells were evaluated. The proportions were scored from 0 to 4 (negative, 0; positive in ≤ 10% of cells, 1; positive in > 10% but ≤ 50% of cells, 2; positive in > 50% but ≤ 75% of cells, 3; and positive in > 75% of cells, 4). The staining intensity was scored from 0 to 3 (absent, 0; weak, 1; moderate, 2; and strong, 3). The two scores were multiplied to obtain the final score (negative, 0; weak, 1–4; moderate, 5–8; and strong, 9–12). The proportion was calculated as the mean percentage of five areas at × 200 magnification.

### Expression related to NEAT1 and ALKBH5

StarBase v2.0 (http://starbase.sysu.edu.cn/starbase2/) [8, 22] was used to predict the relationships between lncRNAs and proteins. RNA sequencing expression data based on The Cancer Genome Atlas (TCGA) and the Genotype-Tissue Expression (GTEx) projects were analyzed with an interactive web server, the Gene Expression Profiling Interactive Analysis (GEPIA) server (http://gepia.cancerpku.cn/index.html) [19].

### Lentiviral vector system, plasmids, and cell transfection

Lentiviruses carrying siRNA sequences targeting human ALKBH5 and NEAT1 were obtained from GeneChem (Shanghai, China). The viruses and Polybrene reagent (Abbott Laboratories, Chicago, IL, USA) were used to infect cells. GC cells were cultured for 72 h in medium containing puromycin for cell screening.

### Scrape motility and transwell invasion assays

A scrape motility assay was used to evaluate cell migration. GCs were plated into culture inserts (Ibidi, Regensburg, Germany). After incubation for 24 h, the inserts were removed. An inverted microscope (XDS-100, Shanghai Caikon Optical Instrument Co., Ltd., Shanghai, China) was used to capture wound monolayer images at 0 and 24 h post wounding.

A transwell assay was performed to evaluate cell invasion. Transwell upper chambers coated with gelatin were plated with GC cells. The lower chambers contained 600 μL of FBS (30%, Costar, Lowell, MA, USA). Methanol and hematoxylin and eosin were used to fix and stain the cells after incubation for 24 h (Sigma-Aldrich, St. Louis, MO, USA). The upper chambers were then removed, and the cells on the surfaces of the lower chambers, the migrated cells, were counted and imaged by microscopy at × 100 magnification in five fields. The average cell number per field represented the number of migrated cells.

### RNA immunoprecipitation

A Magna RIP RNA-Binding Protein Immunoprecipitation Kit (Millipore Corporation) was used to perform RNA immunoprecipitation (RIP) experiments. Antibodies against m^6^A (Abcam) and control proteins were diluted 1:50. Total RNA (the input control) and IgG (the isotype control) were assayed simultaneously with each antibody. RT-PCR was conducted to detect the coprecipitated RNAs.

### Immunofluorescence

Cells were fixed with 4% paraformaldehyde in PBS at room temperature for 10 min and permeabilized with 0.1% Triton-X and 1% BSA in PBS for 30 min at room temperature. DNA staining was performed with 4′,6-diamidino-2-phenylindole (DAPI). NEAT1 (Bioss) was detected with a NEAT1 antibody at 37 °C for approximately 16 h. Imaging was performed using an ECLIPSE Ni microscope (Nikon, Japan).

### Statistical analysis

Statistical analysis was performed with the Statistical Package for the Social Sciences (SPSS; IBM, Armonk, NY, USA) version 19.0. All data are presented as the mean ± SD (standard deviation). Comparisons of the data were conducted with Student’s *t* test when the homogeneity of variance assumption was satisfied between groups; otherwise, the Wilcoxon-signed rank test was used. Multiple groups were compared using one-way analysis of variance (ANOVA). *P* < 0.05 was considered to indicate statistical significance.

## Results

### NEAT1 is overexpressed in human GC

**Fig. 1:**
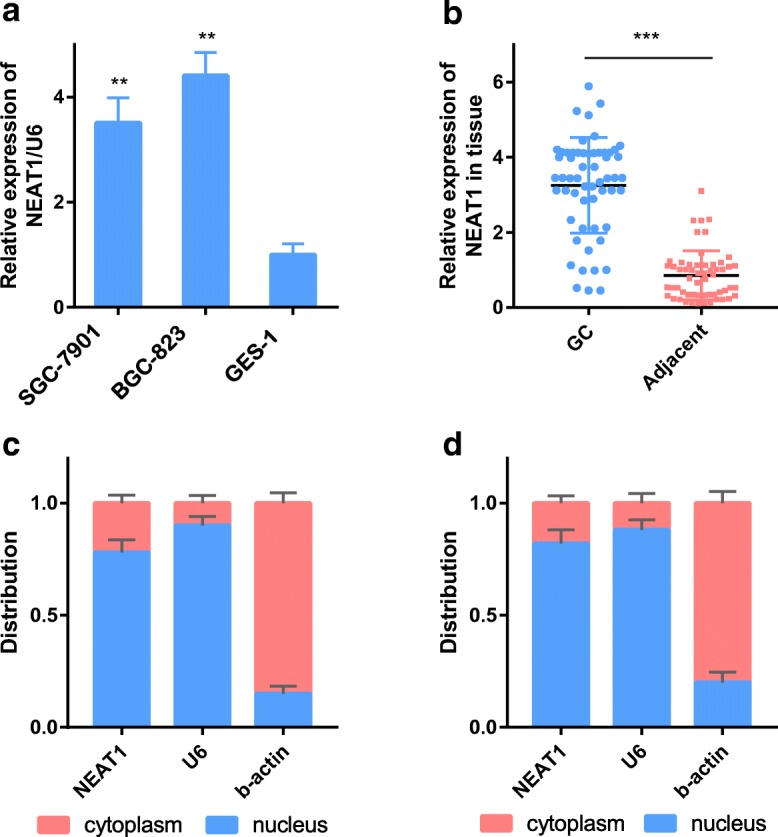
NEAT1 is overexpressed in gastric cancer and is mainly located in the nucleus. **a** NEAT1 expression in GC cell lines (SGC-7901 and BGC-823) and a human normal gastric epithelial cell line (GES-1) as determined by RT-PCR. **b** NEAT1 expression in GC tissue compared with paired adjacent tissue in 57 patients. **c**, **d** The expression level of NEAT1 in the subcellular fractions of SGC-7901 and BGC-823 cells was detected by RT-PCR. U6 and β-actin were used as nuclear and cytoplasmic markers, respectively. U6 was used as a loading control in RT-PCR; *n* = 3, **P < 0.01, ***P < 0.001

The RT-PCR results showed that NEAT1 was overexpressed in BGC-823 and SGC-7901 cells compared with control GES-1 cells (Fig. 1a). We also examined NEAT1 expression using RT-PCR to investigate its roles in GC tissues. The expression levels in tissues corresponding to a total of 57 GC samples were examined and compared with those in their noncancerous counterparts. NEAT1 expression was observed in all GC tissue samples and was upregulated in GC samples compared with adjacent noncancerous samples (Fig. 1b). Moreover, the results of the subcellular distribution assay after nuclear/cytoplasmic RNA fractionation showed that NEAT1 was predominantly located in the nucleus (Fig. 1c, d). Taken together, the results showed that NEAT1 was an overexpressed lncRNA in GC.

### Suppression of NEAT1 expression affected invasion and metastasis of GC cells

**Fig. 2:**
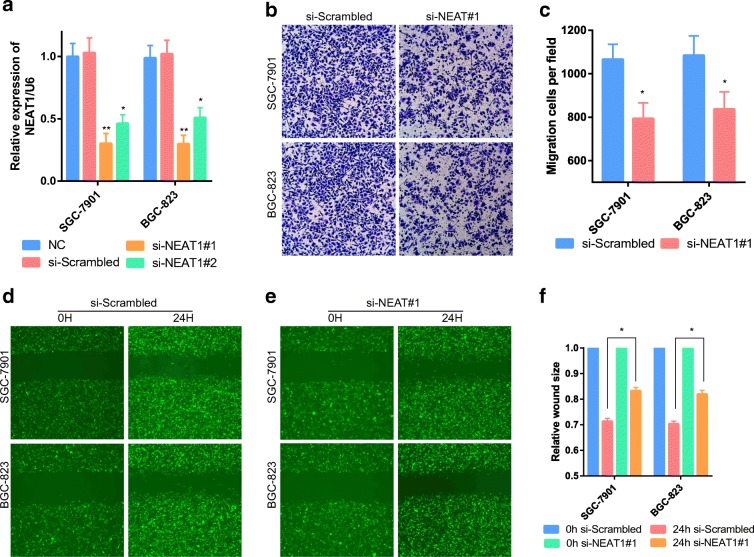
NEAT1 is involved in the cell invasion of gastric cancer. **a** GC cells transfected with si-NEAT1 or NC were assessed by RT-PCR. U6 was used as a loading control in RT-PCR. **b**, **c** Transwell assays with NEAT1-knockdown cells were used to evaluate the role of NEAT1 in invasion. **d**–**f** Scrape motility assays were performed for 24 h with NEAT1-knockdown GC cells. In all figures, × 100 magnification was used. *n* = 3, *P < 0.05, **P < 0.01

To evaluate the biology of NEAT1 in GC, we transfected the GC cell lines SGC-7901 and BGC-823 with NEAT1-specific siRNAs or a control siRNA (si-scrambled). Two siRNAs targeting NEAT1 were tested (si-NEAT1#1 and si-NEAT1#2); si-NEAT1#1 was the most effective in reducing NEAT1 expression and was selected for subsequent research (Fig. 2a). To determine the effect of NEAT1 on cell invasion and metastasis, we performed transwell experiments and scrape motility assays in vitro. Decreases in NEAT1 expression significantly suppressed the invasion and metastasis of GC cells in the transwell (Fig. 2b, c) and scrape (Fig. 2d–f) assays. The observations indicated that NEAT1 is a positive metastatic regulator of GC.

### ALKBH5 is overexpressed in GC and demethylates NEAT1

**Fig. 3:**
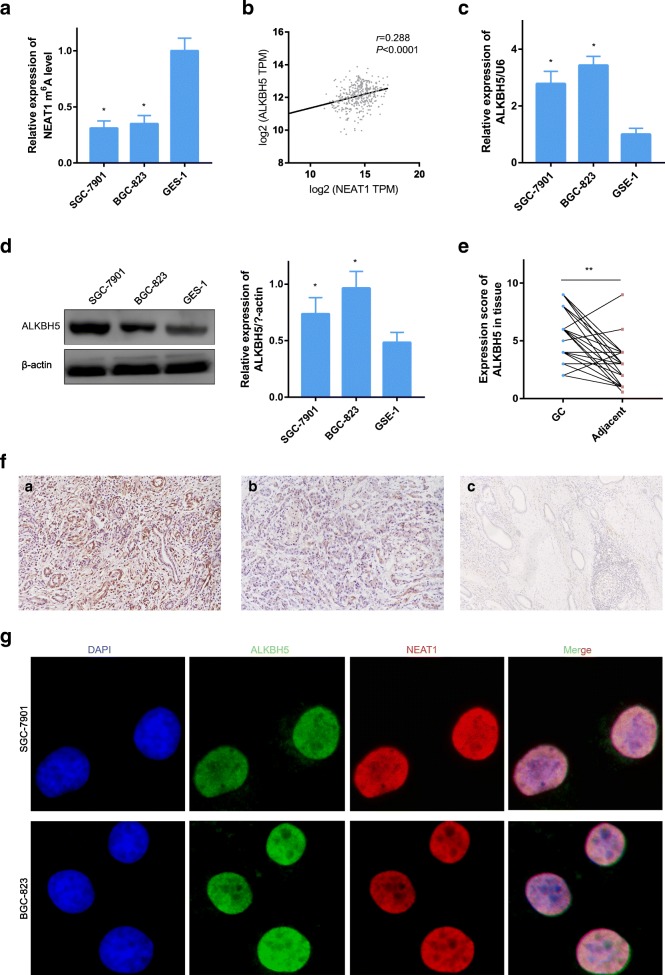
ALKBH5 is overexpressed in GC and colocalizes with NEAT1. **a** RT-PCR followed by RNA immunoprecipitation showed that NEAT1 undergoes m^6^A modification in various gastric cells. **b** ALKBH5 and NEAT1 are coexpressed in GC tissue. **c**, **d** ALKBH5 expression in SGC-7901, BGC-823, and GES-1 cells as determined by RT-PCR and western blot analysis. **e** ALKBH5 expression in GC tissue compared with paired adjacent tissue in 57 patients as determined by IHC. **f** Representative images of ALKBH5 staining in gastric tissue (a, overexpression in GC; b, low expression in GC; c, weak expression in adjacent tissue). **g** Immunofluorescence indicating that ALKBH5 interacts with NEAT1

NEAT1 is enriched in the nucleus [20], similar to FTO and ALKBH5. Therefore, we tested the m^6^A level of NEAT1 in GC cells. The results showed that NEAT1 m^6^A enrichment was lower in SGC-7901 and BGC-823 cells than in GES-1 (Fig. 3a). As predicted by StarBase v2.0, NEAT1 is a target biomarker of ALKBH5. In addition, a positive correlation between NEAT1 and ALKBH5 expression was confirmed by GEPIA (Fig. 3b, *R* = 0.288). Furthermore, we examined the expression of ALKBH5 in cells. Overexpression of ALKBH5 was detected by RT-PCR and western blot analysis in GC cells (Fig. 3c, d). Moreover, to analyze the expression of ALKBH5, 57 paired GC tissues and their noncancerous counterparts were evaluated by immunohistochemistry (IHC). Not surprisingly, the GC tissues had higher scores than the adjacent tissues (Fig. 3e). Typical images of overexpression and low expression are shown in Fig. 3f (a, overexpression in GC; b, low expression in GC; c, weak expression in adjacent tissue). The signals of NEAT1 and m^6^A were found to be colocalized by immunofluorescence and fluorescence microscopy, indicating that NEAT1 underwent *N*^6^-methyladenosine modification (Fig. 3g).

**Fig. 4:**
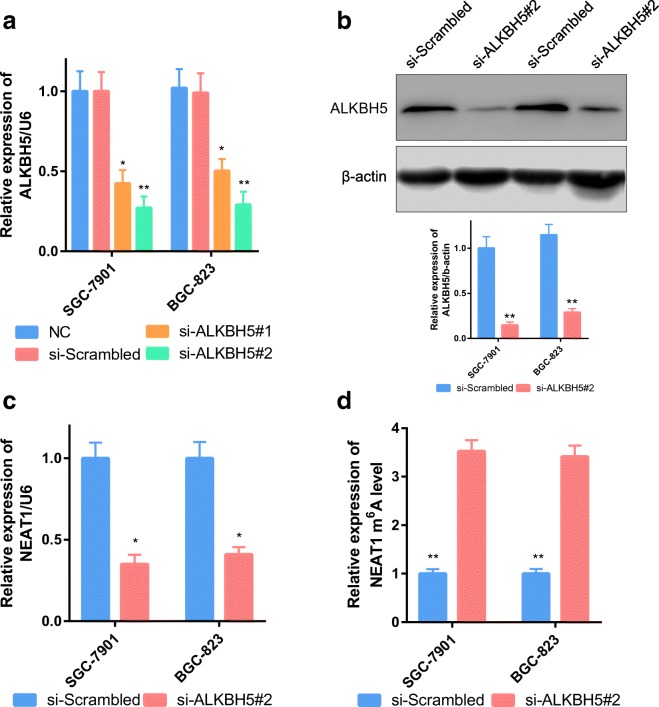
ALKBH5 influences NEAT1 m^6^A modification. **a**, **b** GC cells transfected with si-ALKBH5 or NC were assessed by RT-PCR. U6 and β-actin were used as loading controls in RT-PCR and western blot analysis. **c** NEAT1 was downregulated after ALKBH5 knockdown. **d** NEAT1 methylation was increased after ALKBH5 knockdown

To study the molecular regulation mechanism between ALKBH5 and NEAT1, we knocked down the expression of ALKBH5 in GC cells. Two siRNAs targeting ALKBH5 were tested (si-ALKBH5#1 and si-ALKBH5#2); si-ALKBH5#2 was the most effective in reducing ALKBH5 expression and was selected for subsequent research (Fig. 4a, b). As expected, NEAT1 levels decreased with knockdown of ALKBH5 (Fig. 4c), and NEAT1 m^6^A modification levels were upregulated when the eraser enzyme was absent (Fig. 4d).

### ALKBH5 and NEAT1 affected GC invasion and metastasis by regulating EZH2

**Fig. 5:**
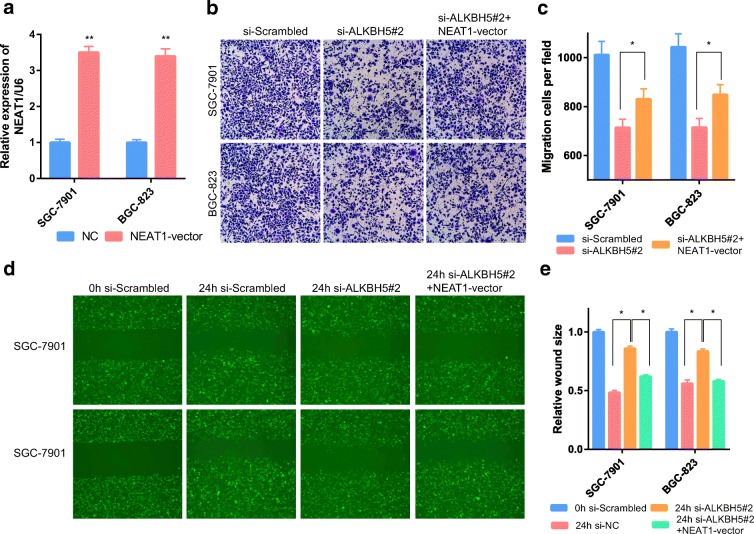
ALKBH5 and NEAT1 affected GC invasion and metastasis. **a** GC cells transfected with NEAT1 vectors were assessed by RT-PCR. U6 was used as a loading control in RT-PCR. **b**, **c** Transwell assays were used to evaluate the association of NEAT1 and ALKBH5 during invasion in ALKBH5-knockdown and ALKBH5-knockdown + NEAT1-overexpressing GC cells. **d**, **e** Scrape motility assays were performed for 24 h with ALKBH5-knockdown and ALKBH5-knockdown + NEAT1-overexpressing GC cells. In all figures, × 100 magnification was used. *n* = 3, *P < 0.05

**Fig. 6:**
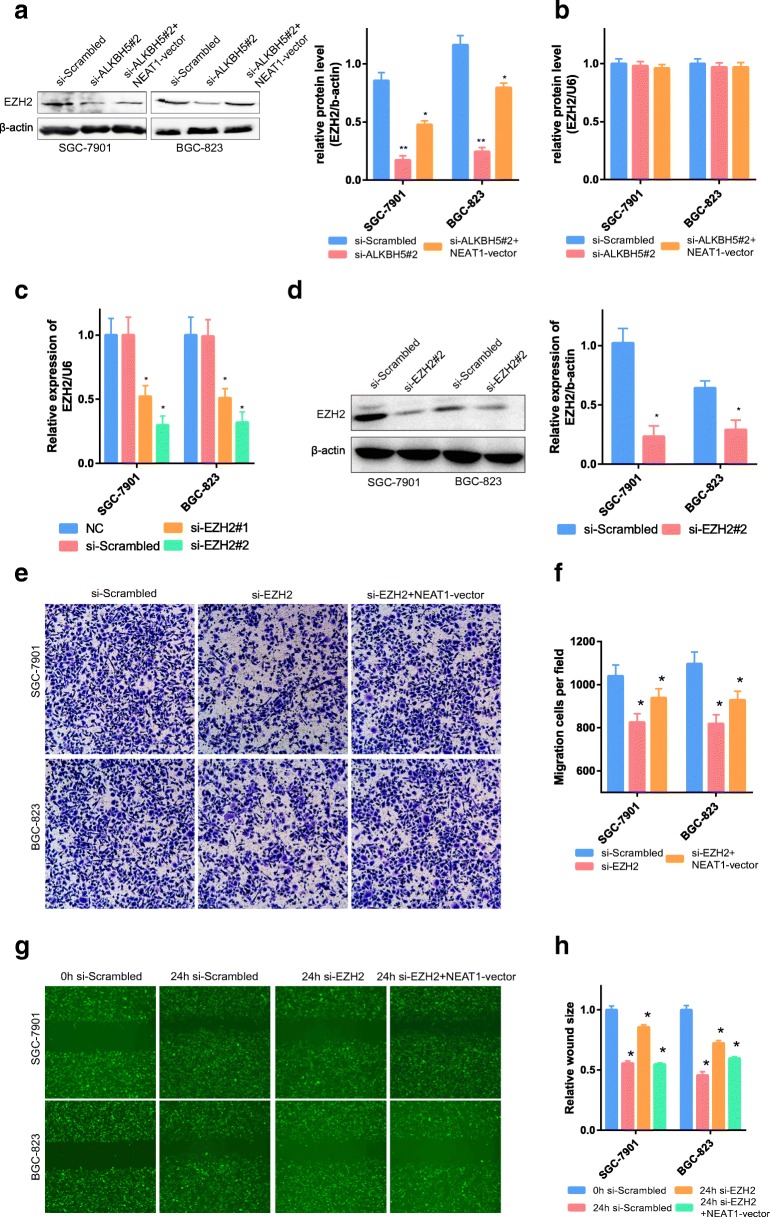
EZH2 and NEAT1 affected GC invasion and metastasis. **a** The expression of EZH2 protein was affected by NEAT1 and ALKBH5. **b** The mRNA level of EZH2 was not affected by NEAT1 or ALKBH5. **c**, **d** GC cells transfected with si-EZH2 or NC were assessed by RT-PCR. U6 and β-actin were used as loading controls in RT-PCR and western blot analysis. **e**, **f** Transwell assays were used to evaluate the role of EZH2 in invasion in EZH2-knockdown and EZH2-knockdown + NEAT1-overexpressing GC cells. In all figures, × 100 magnification was used. **g**, **h** Scrape motility assays were monitored for 24 h in EZH2-knockdown and EZH2-knockdown + NEAT1-overexpressing GC cells. In all figures, × 100 magnification was used

To study the biological function of ALKBH5 in GC and to further verify that it is closely related to NEAT1, we stably suppressed ALKBH5 expression and overexpressed NEAT1 (Fig. 5a). Transwell experiments and scrape motility assays were performed in vitro. These experiments showed that reductions in ALKBH5 expression significantly inhibited the invasion and metastasis of GC cells (Fig. 5b–e); this inhibition was partially attenuated by overexpression of NEAT1 (Fig. 5b–e). NEAT1 can function as a scaffold by interacting with EZH2 (a subunit of the polycomb repressive complex) to regulate the expression of downstream genes of EZH2, and this regulation is associated with invasion and metastasis [2]. We also examined the expression of EZH2 in GC cells. The expression level of EZH2 could be inhibited by si-ALKBH5. In addition, this inhibition could be partly reversed by overexpression of NEAT1 (Fig. 6a). However, the mRNA level of EZH2 was not regulated by NEAT1 expression (Fig. 6b). This finding indicates that NEAT1 regulated EZH2 at the posttranscriptional level.

To demonstrate the effect of EZH2 on cell invasion and metastasis, we performed corresponding gene manipulation experiments on EZH2. EZH2 mRNA could be inhibited by two siRNAs; si-EZH#2 was the most effective in reducing EZH2 expression (Fig. 6c, d). In addition, transwell experiments and scrape motility assays also showed that reductions in EZH2 inhibited the invasion and metastasis of GC cells (Fig. 6, *P* < 0.01); this decrease was partially attenuated by overexpression of NEAT1 (Fig. 6e–h, *P* < 0.01). These results indicated that the abnormal invasion and metastasis of GC were partly attributable to the dysregulation of EZH2 controlled by ALKBH5 and NEAT1.

**Fig. 7:**
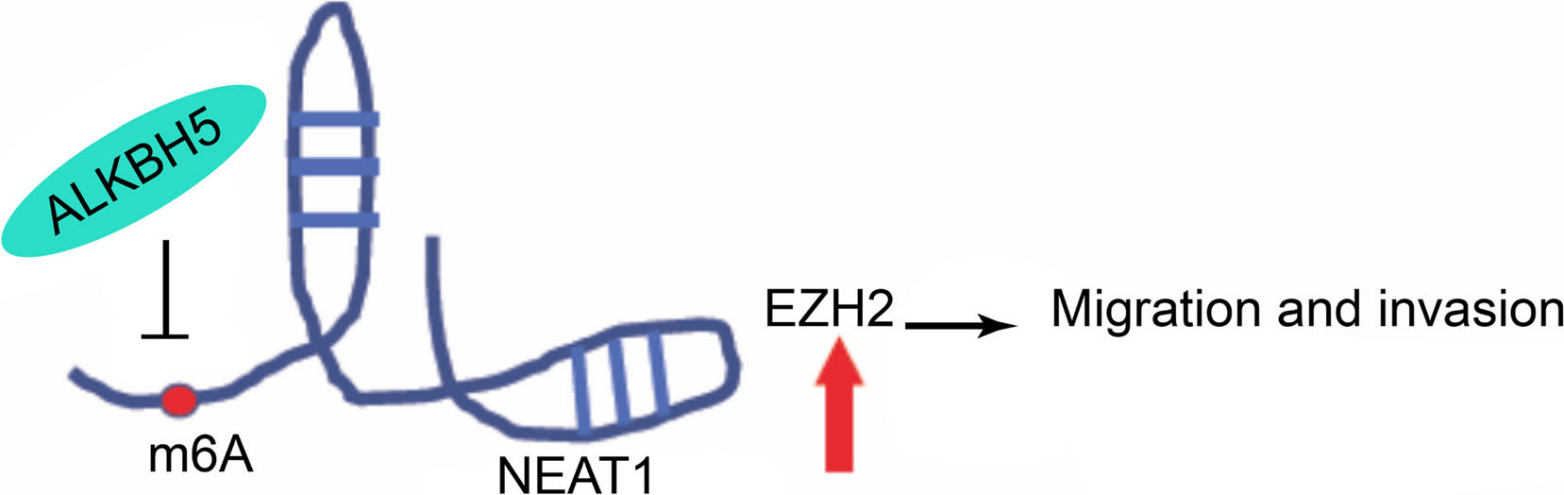
Illustrative model showing the proposed mechanism by which NEAT1 promotes invasion and metastasis in GC through the demethylase ALKBH5

In summary, ALKBH5 influences the expression of NEAT1 through demethylation, and overexpression of NEAT1 can subsequently lead to overexpression of EZH2 and eventually to a corresponding malignant phenotype (Fig. 7).

## Discussion

Dysregulation of lncRNAs is related to the formation, progression, metastasis, and prognosis of various tumors [15]. The subcellular localization of an lncRNA determines its function. Cytoplasmic lncRNAs affect the transcription, translation, and stability of mRNAs, and nuclear lncRNAs are associated with transcriptional and epigenetic modulation [13]. One well-known lncRNA, NEAT1, is transcribed from the tumor syndrome multiple endocrine neoplasia (MEN) family type 1 locus on chromosome 11q13.1. Although NEAT1 can be found in the cytoplasm, it is mainly enriched in the nucleus [1]. NEAT1 is overexpressed in various cancers [18], and upregulation of NEAT1 is positively correlated with the poor overall survival of these cancers [28]. NEAT1 exerts its carcinogenic effects through three main mechanisms: it acts as a miRNA sponge to antagonize the interactions between tumor suppressor miRNAs and target mRNAs [18, 28]; it acts as a scaffold, binding with EZH2 to promote the expression of downstream genes of EZH2 [2]; and it promotes DNA methylation by inhibiting the expression of miR-129 [11]. However, the epitranscriptional modification of NEAT1 remains unclear. Our results identified that NEAT1 is upregulated in GC and that overexpressed NEAT1 can act synergistically with EZH2 to promote invasion and metastasis.

It is widely recognized in the nascent field of epigenetics that the reversible processes of m^6^A modification control and determine cell growth and differentiation [24]. However, the role of m^6^A in cancers is unclear. Zhang et al. [25] revealed that overexpression of ALKBH5, which decreases the FOXM1 m6A levels, results in increased FOXM1 expression and eventually in glioblastoma. However, another report noted that increases in METTL3 m^6^A levels enhance the translation of target mRNAs (RGFR and TAZ), promoting the formation of lung cancer [9]. Two independent research groups have found that METTL14 exerts opposing biological functions in hepatic carcinoma [3, 12]. Unfortunately, the importance of lncRNA methylation in tumorigenesis and development remains unclear. From our results, we found that NEAT1 underwent m^6^A methylation in GC. Additionally, the m^6^A eraser ALKBH5 could downregulate NEAT1 m^6^A levels. The expression of ALKBH5 and NEAT1 was positively correlated. With decreases in NEAT1 methylation, NEAT1 was upregulated, which promoted the malignant phenotype of GC. Because NEAT1 can be used as a scaffold to affect the expression of EZH2 and affect the invasion and metastasis of tumors, we also verified the mechanisms in this study and obtained similar conclusions as those obtained in previous studies.

Although we identified and verified the importance of NEAT1 methylation in the invasion and metastasis of GC using molecular biological approaches, the present study had some limitations. First, the study did not identify a direct mechanism. Second, the expression of NEAT1 and its functions in GC should be validated in in vivo experiments.

## Abbreviations

m^6^A: *N*^6^-methyladenosine
lncRNA: long noncoding RNA
GC: Gastric cancer
ALKBH5: alkylation repair homolog protein 5
NEAT1: nuclear paraspeckle assembly transcript 1
EZH2: a subunit of the polycomb repressive complex
HGP: Human Genome Project
NGS: next-generation sequencing
METTL3: methyltransferase-like 3
METTL14: methyltransferase-like 14
FTO: fat mass and obesity-associated protein
GEPIA: Gene Expression Profiling Interactive Analysis
TCGA: The Cancer Genome Atlas
GTEx: Genotype-Tissue Expression
RIP: RNA immunoprecipitation
